# Psychometric properties of the Arabic version of the Age-Friendly Cities and Communities Questionnaire (AFCCQ-Arabic)

**DOI:** 10.3389/fsoc.2025.1640423

**Published:** 2025-11-18

**Authors:** Hanan AboJabel, Liat Ayalon, Jeroen Dikken, Joost van Hoof

**Affiliations:** 1 The Paul Baerwald School of Social Work and Social Welfare, Mount Scopus Jerusalem, The Hebrew University of Jerusalem, Jerusalem, Israel; 2 Louis and Gabi Weisfeld School of Social Work, Bar Ilan University, Ramat Gan, Israel; 3 Research Group of Urban Ageing, Faculty of Social Work and Education, The Hague University of Applied Sciences, The Hague, Netherlands; 4 Faculty of Health, Nutrition and Sport, The Hague University of Applied Sciences, The Hague, Netherlands; 5 Department of Systems Research, Faculty of Spatial Management and Landscape Architecture, Wrocław University of Environmental and Life Sciences, Wrocław, Poland

**Keywords:** age-friendly cities and communities, older adults, Arab minority, psychometric validation, Arabic AFCCQ

## Abstract

**Background and objective:**

The World Health Organization has promoted the concept of Age-Friendly Cities and Communities (AFCC) as a response to global population aging. This approach aims to adapt physical and social environments to the needs of older adults, particularly in the context of ongoing urbanization. The Age-Friendly Cities and Communities Questionnaire (AFCCQ) was designed to assess older adults’ perceptions of age-friendliness in their communities but has not yet been validated in Arabic. This study aimed to translate, culturally adapt, and evaluate the psychometric properties of the Arabic version of the AFCCQ (AFCCQ-Arabic), and to assess how older Arabs in Israel perceive the age-friendliness of their communities. This population is an ethnic minority facing dual challenges given its age and minority status.

**Methods:**

A cross-sectional sample of 202 Arab adults aged 60 and above was recruited through convenience sampling. Data were collected through face-to-face interviews using the AFCCQ-Arabic, which includes 23 items across nine domains.

**Results:**

The AFCCQ-Arabic demonstrated overall acceptable psychometric properties among older Arabs in Israel. Face and content validity were supported. Construct validity was assessed using Confirmatory Factor Analysis (CFA). The original model demonstrated good fit indices (CFI = 0.922; TLI = 0.900; RMSEA = 0.064; SRMR = 0.072). Most domains showed acceptable Composite Reliability (CR) values, indicating good internal consistency. However, item 3 demonstrated a low factor loading and reduced the CR value of the social participation domain. Additionally, the civic participation and employment domain showed low reliability (CR = 0.28). Domain-level scores revealed diverse perceptions of age-friendliness: high scores in respect and social inclusion, housing, civic participation and employment, and community support and health services; moderate scores in social participation, and communication and information; and low scores in transportation, outdoor spaces and buildings, and financial situation.

**Conclusion:**

Despite some psychometric limitations, the AFCCQ-Arabic was found to be reliable, valid, and culturally appropriate for use among older Arab adults in Israel. The variation in domain scores indicates service, infrastructure, and economic security gaps in Arab communities, underscoring the need for targeted policy interventions to promote equitable aging.

## 1 Introduction

Over the past two decades, the World Health Organization (WHO) has been promoting the concept of age-friendly cities and communities (AFCC) as a strategic response to global population aging, particularly in the context of ongoing urbanization. These initiatives are designed to help older adults remain active, involved, and respected within society by creating environments that support their evolving circumstances (Sánchez-González et al., 2020; van Hoof and Marston, 2021; World Health Organization, 2015; World Health Organization, 2007; Woolrych et al., 2022). An age-friendly cities and community is characterized by its ability to foster positive aging through supportive physical and social surroundings. It adjusts to the diverse needs, preferences, and capacities of older people, ensures their inclusion in civic life, protects those who are most at risk, acknowledges their societal contributions, and delivers services, infrastructure, and policies that reflect these commitments (World Health Organization, 2007). As the AFCC movement has gained momentum, with over 1,700 members worldwide, research on both its theoretical underpinnings and practical implementation has expanded significantly (Rémillard-Boilard et al., 2021; Steels, 2015; Torku et al., 2021). A growing body of work has focused on older adults’ own perceptions of their environments, recognizing that the success of such initiatives depends not only on policy and infrastructure but also on how older people experience and evaluate their everyday surroundings (Chui et al., 2022; Hussein et al., 2024; Kim et al., 2022; van Hoof et al., 2022). Consequently, increasing efforts have been made to develop validated instruments that assess older adults’ attitudes and experiences regarding AFCC.

### 1.1 The Age-Friendly Cities and Communities Questionnaire

One of the key instruments developed for this purpose is the Age-Friendly Cities and Communities Questionnaire (AFCCQ), created by Dikken et al. (2020). The questionnaire was originally developed in Dutch and designed in accordance with the COnsensus-based Standards for the selection of health Measurement INstruments (COSMIN) guidelines. Its structure is based on a conceptual model grounded in the World Health Organization’s (WHO) framework for AFCC. The AFCCQ underwent a comprehensive validation process that included qualitative assessments such as face validity, readability, and content validity, as well as psychometric testing covering structural validity, convergent validity, and internal consistency. The final version comprises 23 items across nine key domains: housing, social participation, respect and social inclusion, civic participation and employment, communication and information, community support and health services, outdoor spaces and buildings, transportation, and financial situation. Notably, the financial domain was added to reflect the growing recognition of financial security as a critical component of age-friendliness.

The AFCCQ has been translated into British English through a forward–backward translation procedure and has since been adapted and validated in several other languages, including Turkish (Özer et al., 2023), Romanian (Ivan et al., 2024), Polish (Perek-Białas et al., 2024), Japanese (Yamada et al., 2023), Portuguese (Barata et al., 2026), Hebrew (Ayalon et al., 2024), Russian (Ziganshina et al., 2025), Italian (Bertani et al., 2025), German (Grenz et al., 2025), Macedonian and Albanian (Pavlovski et al., 2024). It has also been applied in studies in Australia (Wasserman et al., 2025) and New Zealand (Piercy et al., 2025). This extensive linguistic and cultural validation allows for robust cross-cultural comparisons and the identification of both universal and context-specific aspects of age-friendliness.

### 1.2 The need for Arabic validation in the Israeli context

Despite the growing body of research in the field, the AFCCQ has not yet been validated in Arabic, representing a notable gap given that Arabic is spoken by approximately 491 million people worldwide, making it the fifth most spoken language globally (Boshers, 2025). Arabic is also one of the six official languages of the United Nations and is used by major international organizations, including the WHO. This validation is especially important in the Israeli context, where Arabs comprise about one-fifth of the population (Israel Central Bureau of Statistics, 2024). As a minority group, they face unique cultural, social, and political circumstances that may shape their experiences of aging and their perceptions of the living environment.

Arabs are the largest minority group among Israel’s citizens, with approximately 85% identifying as Muslim, alongside smaller groups such as Christians, Druze, and others (Israel Central Bureau of Statistics, 2022). Older Arabs constitute about 5% of the Arab population, compared to 12% in the Jewish population, reflecting a significantly younger demographic profile. The vast majority of Arabs in Israel reside in Arab towns and villages, mainly in the Galilee region of northern Israel and the Triangle region, and are experiencing a process of accelerated urbanization (Khamaisi, 2013; Shmueli and Khamaisi, 2015). Only about 8% live in mixed cities where Jews and Arabs share residential space (Ron et al., 2022).

The differences between Jews and Arabs in Israel extend beyond language and geography; they are also deeply rooted in social and cultural structures. The Arab society tends to be more collectivistic and traditional, placing strong emphasis on family ties and intergenerational relationships (AboJabel and Abo-Rass, 2025). Older adults have historically held central and respected roles within Arab communities, such as offering family guidance, making community decisions, and mediating conflicts. However, processes of modernization have increasingly undermined their traditional status and authority (Azaiza and Kraituro, 2010; Manor, 2020).

In addition to cultural differences, older Arabs face multiple social and health disparities. For example, more than half (approximately 58%) of older Arabs live below the poverty line, and about one third (32.5%) of the Arab population have completed no more than 4 years of formal education (Myers-Joint-Brookdale Institute, 2023; Nathanson et al., 2018). Older Arabs also experience higher rates of chronic diseases, disability, mental health challenges, and dementia compared with their Jewish counterparts (AboJabel et al., 2015; Dwolatzky et al., 2017; Saabneh, 2016; Saabneh, 2015; Werner et al., 2015). Although healthcare and welfare services are officially available for all citizens in Israel, older Arabs make less use of them. Contributing factors include language difficulties, limited digital literacy, and cultural reservations toward formal welfare systems. Another significant factor is their geographic location: 44.5% of Arabs live in Israel’s periphery, compared to just 13.4% of Jews. Peripheral areas often suffer from shortages of healthcare and social services, and existing services tend to be of lower quality than those in central regions (Naim et al., 2017; Chernichovsky et al., 2017; Shibli et al., 2021; Vitman-Schorr and Khalaila, 2022; Werner and Tur-Sinai, 2024).

Residential patterns also influence older adults’ experiences. Most Arabs in Israel reside in self-built homes located in semi-urban areas. Self-built private houses remain the dominant housing type; however, space constraints and planning restrictions have led many families to expand vertically, often by adding additional floors or housing units on existing plots. Despite a continuing cultural preference for detached homes, demographic and economic pressures have contributed to a gradual shift toward apartment living in increasingly dense residential environments (Khamaisi, 2013; Shmueli and Khamaisi, 2015).

*Study aims*: These intersecting factors highlight the importance of developing culturally sensitive and linguistically appropriate tools to assess age-friendliness among older Arabs in Israel. To date, no validated Arabic version of the AFCCQ exists, and little is known about how this population perceives and evaluates their cities and communities in relation to age-friendly principles. Gaining insight into their experiences is essential for shaping inclusive planning of living environments, healthcare, welfare services, and public policy. Therefore, the current study aims to: (1) translate the AFCCQ into Arabic and evaluate its psychometric properties, including reliability and validity; and (2) examine how older Arabs in Israel perceive and evaluate the age-friendliness of their living environments.

## 2 Methods

### 2.1 Design and procedure

**Figure 1:**
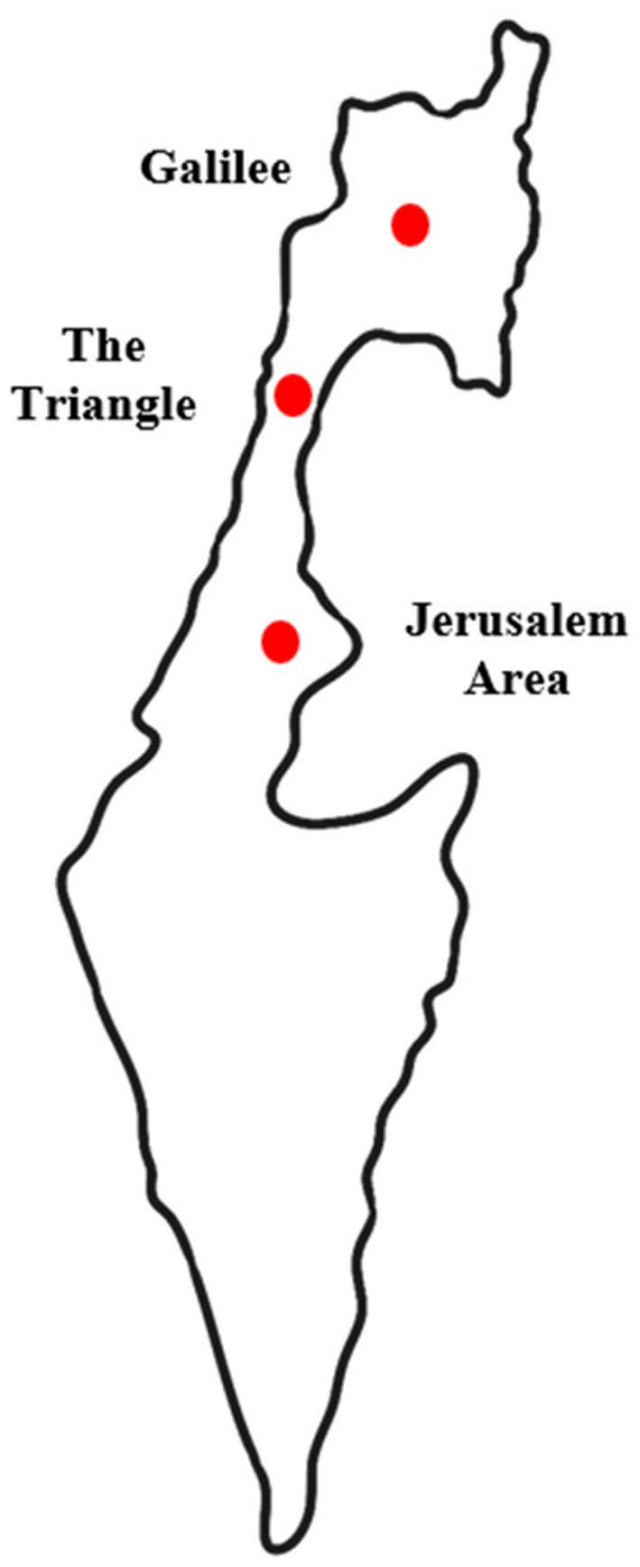
Map of Israel highlighting the three regions included in the study: Galilee, Triangle Area, and Jerusalem Area. Source: Shutterstock, Stock Vector ID 405992995.

A cross-sectional and anonymous survey was conducted among 202 Arab adults aged 60 years and older who are citizens of Israel. The sample size was determined based on previous studies that used samples of at least 100 participants to validate measurement scales (Anthoine et al., 2014). In addition, following common recommendations for studies conducting factor analysis, a sample of approximately 200 participants is considered sufficient to perform factor analysis and estimate structural models (Kline, 1994). Furthermore, according to the guidelines of Hair et al. (2019), a minimum ratio of five participants per item (5:1) is recommended. In the current study, there were 23 items and 202 participants, resulting in a ratio of about nine participants per item (9:1), which exceeds the minimum threshold and indicates an adequate sample size for conducting a reliable and valid factor analysis. A convenience sampling method was utilized, and participants were recruited through personal contacts, senior day centers, and various community settings in 14 Arab cities and villages in the Galilee (northern Israel), the Triangle region (central Israel), and the Jerusalem area (see Figure 1). Convenience sampling was chosen due to difficulties in reaching a representative population, including language barriers, low socioeconomic status, limited education, low digital literacy, and limited trust in institutions and researchers, factors often observed among minority groups worldwide (Bonevski et al., 2014). Data were collected through face-to-face interviews conducted by the first author (H. A. J.), a gerontologist, and two trained Arabic-speaking research assistants. Interviews were held in senior day centers, community centers or, according to participants’ preferences, in their homes, and each lasted approximately 15–20 min. All participants provided informed consent prior to participation. Data collection took place between August 2024 and February 2025, with temporary pauses in areas affected by security escalations. All research activities followed the safety guidelines of the Israeli Home Front Command, the national civil defense authority operating within the Israel Defense Forces (IDF). The Home Front Command provides public safety instructions, issues emergency alerts, and prepares the civilian population for emergencie.^1^ Following these guidelines helped ensure the safety of both the participants and the research team. Since data collection occurred during a period of heightened security tension, the interviewers remained attentive to possible signs of psychological distress among participants and were instructed to stop the interview if necessary. In practice, no interviews had to be stopped.

## 3 Measures

### 3.1 The Age-Friendly Cities and Communities Questionnaire

The AFCCQ, developed by Dikken et al. (2020), comprises 23 items organized into nine domains: housing (2 items), social participation (4 items), respect and social inclusion (2 items), civic participation and employment (2 items), communication and information (2 items), community support and health services (5 items), outdoor spaces and buildings (2 items), transportation (2 items), and financial Situation (2 items). In the current analysis, we recalculated domain scores using a recoded scoring system based on the guidelines used in previous applications of the AFCCQ. Specifically, the Likert responses were recoded as follows: 1 = −2, 2 = −1, 3 = 0, 4 = +1, and 5 = +2. Following this recoding, the average score for each domain was calculated, resulting in possible mean values ranging from −2 to +2. A mean score closer to +2 indicates higher satisfaction with the domain, whereas a score closer to −2 reflects lower satisfaction.

### 3.2 Background characteristics

Participants were asked to report their age, gender, years of education, place of residence, and the number of years they had lived in their current location. They were also asked to provide information regarding their housing type (owner-occupied or private rental) and living arrangement (living alone or with others). In addition, participants were asked whether they receive care at home (such as cleaning, personal care, or caregiving assistance), whether they have a chronic illness, and whether they use mobility aids. Responses to these three items were recorded as “yes” or “no.” Finally, participants rated their perceived quality of life on a scale from 0 to 10, with 0 indicating the lowest and 10 the highest perceived quality of life. Consistent with previous studies (Huang et al., 2025), scores were interpreted as follows: low (0–5), moderate (6–8), and high (9–10).

### 3.3 Statistical analyses

Descriptive statistics [means, standard deviations (SD), frequencies, and percentages] were used to summarize participant characteristics and to present item- and dimension-level distributions of the AFCCQ. A small proportion of missing values (0.6%) was identified in the questionnaire items and replaced with the mean of the corresponding variable (Hair et al., 2019; Rioux and Little, 2021). To evaluate the performance of the AFCCQ items, distributional properties were examined using measures of asymmetry (skewness) and kurtosis to assess univariate normality, with skewness values within ±3 and kurtosis within ±10 considered acceptable indicators of normal distribution (Kline, 2016). Multivariate normality was examined using Mahalanobis D^2^ statistics, where values exceeding the χ^2^ critical threshold at *p* < 0.001 (Hair et al., 2019) indicated potential outliers. Construct validity was assessed using confirmatory factor analysis (CFA), and items with standardized factor loadings ≥ 0.40 were retained. Model fit was evaluated according to established criteria: chi-square to degrees of freedom ratio (χ^2^/df < 3), root mean square error of approximation (RMSEA < 0.08) with a 90% confidence interval whose upper limit did not exceed 0.08, standardized root mean square residual (SRMR < 0.08), comparative fit index (CFI > 0.90), and Tucker–Lewis index (TLI > 0.90) (Kline, 2016; Moss et al., 2015). Internal consistency was examined using composite reliability (CR), with values above 0.70 indicating adequate reliability (Hair et al., 2019). All analyses were conducted using SPSS version 30 and Amos version 26.

### 3.4 Ethical considerations

The research protocol was approved by the ethics committee of the Paul Baerwald School of Social Work and Social Welfare at the Hebrew University of Jerusalem (Approval No. 14082024).

## 4 Results

### 4.1 Background characteristics of the participants

As shown in Table 1, the average age of the total sample was approximately 74 years (SD = 6.13). The majority of participants were women (65%) and most resided in northern Israel (70%). On average, participants had been living in their current place of residence for about 64 years (SD = 17.64). Additionally, an overwhelming majority (98%) owned their homes, and 70% lived alone. Most participants (60%) reported receiving assistance at home, such as cleaning services, personal care, or caregiving support. Furthermore, 61% reported coping with chronic illnesses, and 43% used mobility aids for walking. Finally, participants rated their quality of life at an average level (Mean = 6, range = 0–10).

**Table 1:** Background characteristics of the study participants (*n* = 202).

Variable	(%)/Mean (SD, range)
Mean age (SE, range)	74.32 (6.13, 60–93)
Gender (%)	
Women	65.3
Men	34.7
Mean years of education (SE, range)	7.76 (5.21, 0–20)
Region of residence (%)	
North	69.8
Central (Triangle Area)	10.4
Jerusalem district	19.8
Average years of residence in the place of residence (SE, range)	64.03 (17.64, 2–88)
Type of housing (%)	
Owner-occupied home	98.5
Private rent	1.5
Living alone vs. with others (%)	
Alone	70.1
With others	29.9
Receive care at home (%)	
No	39.2
Yes	60.8
Chronic illness (%)	
No	39.2
Yes	60.8
Mobility aid (%)	
No	57.2
Yes	42.8
Mean quality of life (SD, range)	5.93 (2.49, 0–10)

### 4.2 Translation and face validation of the AFCCQ-Arabic

For the purpose of this study, the questionnaire was translated from English into Arabic using the back-translation method by two independent experts, following established guidelines (Perestelo-Pérez et al., 2021). Face validity was further assessed through face-to-face interviews with six older adults who were not part of the final study sample. These participants were asked to evaluate the relevance of each item using a 4-point scale, where 1 indicated “totally irrelevant” and 4 indicated “highly relevant.” At the end of the interview, participants were also asked whether the questions were well formulated and whether they understood them. Most items were rated as highly relevant (score of 4) by all participants. However, two items (Items 1 and 2) received lower ratings from three participants, as the gap between spoken Arabic and the literary Arabic (Arabic fusha) used in the questionnaire led to difficulties in understanding the questions. To enhance clarity and ensure accurate understanding, these items were revised to include both the literary Arabic term and its commonly used spoken equivalent, thereby preserving the intended meaning while improving comprehensibility for respondents.

### 4.3 Content validation of the AFCCQ-Arabic

To evaluate the content validity of the AFCCQ-Arabic, seven Arab experts independently assessed the cultural and social relevance of the questionnaire items and the extent to which they reflect the construct being measured among the Arab population. The group included three academic researchers whose work focuses on aging in the Arab population in Israel, three professionals with extensive practical experience working with older adults in Arab communities, and one expert in environmental development. The experts were selected through purposive sampling.

Each expert independently rated the relevance of each item using a 4-point scale ranging from 1 (“Not relevant at all”) to 4 (“Highly relevant”). Most items received the highest score of 4 from all experts, while a few items were rated as 3 (“Largely relevant”) by one or two of the experts. These results indicate a strong level of agreement regarding the cultural and social appropriateness of the items. Consequently, all items were retained in the final version of the questionnaire. However, based on expert feedback, minor wording adjustments were made to several items, and two items (Items 20 and 21) were culturally adapted. In the original version, these items referred to the availability of public transportation and included examples such as busses and the tram. For example: “Item 20: I can easily get on the bus or tram in my neighborhood”; “Item 21: The bus and tram stops in my neighborhood are easy to reach and use.” Since tram systems do not exist in Arab localities in Israel, experts recommended removing the reference to the tram, as participants might not be familiar with this mode of transportation. The items were revised accordingly: “Item 20: I can easily get on a bus in my neighborhood”; “Item 21: The bus stops in my neighborhood are easy to reach and use.”

### 4.4 Item descriptive statistics

Descriptive statistics for the items of the AFCCQ-Arabic scale, including mean, standard deviation, skewness and kurtosis, are summarized in Table 2. As shown in Table 2, the highest domain-level score was observed in respect and social inclusion (Mean = 1.49, SD = 0.95). This result suggests that older adults experienced low levels of ageism and felt positively regarded within their communities. Other relatively high scores were found in housing (Mean = 1.00, SD = 1.27), civic participation and employment (Mean = 0.87, SD = 0.92), and community support and health services (Mean = 0.68, SD = 0.91), suggesting favorable perceptions in these areas. Moderate ratings were found in social participation (Mean = 0.30, SD = 1.02) and communication and information (Mean = 0.08, SD = 1.43), while the lowest means emerged in financial Situation (Mean = −0.08, SD = 1.39), transportation (Mean = −0.38, SD = 1.34), and outdoor spaces and buildings (Mean = −1.06, SD = 1.23), indicating greater dissatisfaction with infrastructure and economic conditions.

**Table 2:** Descriptive statistics of items in the AFCCQ-Arabic.

English Item Content	Arabic Item Content	Mean (SD, Rang)	Skewness (SE=0.17)	Kurtosis (SE=0.34)
Domain 1: Housing	مجال 1: الإسكان			
1. My house is accessible to me	1. بيتي متاح أو مُهيّأ لي	0.80 (1.39, ±2)	-0.93	-0.53
2. My house is accessible to the people who come to visit me	2. بيتي متاح أو مُهيّأ بالنسبة للأشخاص الذين يأتون لزيارتي	1.20 (1.13, ±2)	-1.70	2.23
Total		1.00 (1.27)		
Domain 2: Social participation	مجال 2: المشاركة الاجتماعية			
3. There are enough opportunities to meet people in my neighbourhood	3. هناك فرص كافية للالتقاء بالناس في حارتي	0.27 (1.46, ±2)	-0.39	-1.26
4. Activities and events are organised in places that are accessible to me	4. يتم تنظيم الأنشطة والفعّاليّات في الأماكن التي من السهل الوصول إليها بالنسبة لي	0.30 (1.45, ±2)	-0.29	-1.33
5. The information about activities and events is enough and also suitable for me	5. المعلومات عن النشاطات والفعاليات كافية ومناسبة أيضًا بالنسبة لي	0.26 (1.41, ±2)	-0.29	-1.28
6. I find the range of events and activities sufficiently varied	6. أرى أنّ مدى النشاطات والفعاليات متنوّع بما فيه الكفاية	0.39 (1.37, ±2)	-0.48	-1.02
Total (with item 3)		0.30 (1.02)		
Total (Without item 3)		0.31 (1.18)		
Domain 3: Respect and social inclusion	مجال 3: الاحترام والإدماج الاجتماعي			
7. I sometimes get annoying or negative remarks because of my age^1^	7. أحيانًا أحصل على ملاحظات مزعجة أو سلبيّة بسبب عمري	1.58 (0.91, ±2)	-2.54	6.01
8. I sometimes face discrimination because of my age^1^	8. أواجه أحيانًا التمييز بسبب عمري	1.41 (1.13, ±2)	-1.80	1.84
Total		1.49 (0.95)		
Domain 4: Civic participation and employment	مجال 4: المشاركة المدنية والتوظيف			
9. I have enough opportunities to interact with younger generations	9. لديّ فرص كافية للقاء الأجيال الشابة	0.55 (1.27, ±2)	-0.63	-0.63
10. I feel like a valued member of society	10. اشعر بأنني عضو يحظى بالتقدير في المجتمع	1.19 (1.12, ±2)	-1.54	1.62
Total		0.87 (0.92)		
Domain 5: Communication and information	مجال 5: الاتصال والمعلومات			
11. Printed and digital information from the municipality and other social institutions is easy to read in terms of font and size	11. يسهل قراءة المعلومات المطبوعة والرقميّة من البلديّة والمؤسّسات الاجتماعيّة الأخرى من حيث الخطّ والحجم	0.19 (1.54, ±2)	-0.21	-1.48
12. Printed and digital information from the municipality and other social institutions is written in understandable language	تتمّ كتابة المعلومات المطبوعة والرقميّة من البلدية والمؤسّسات الاجتماعيّة الأخرى بلغة مفهومة 12.	-0.01 (1.60, ±2)	-0.04	-1.63
Total	12. تتمّ كتابة المعلومات المطبوعة والرقميّة من البلدية والمؤسّسات الاجتماعيّة الأخرى بلغة مفهومة	0.08 (1.43)		
Domain 6: Community support and health services	مجال 6: دعم المجتمع والخدمات الصحية			
13. The supply of care and welfare in my city is enough for me	13. توفير الرعاية والرفاه في مدينتي كافٍ بالنسبة لي	0.45 (1.33, ±2)	-0.47	-1.03
14. When I am ill, I receive the care and help I need	14. عندما أكون مريضًا، أتلقّى الرعاية والمساعدة التي أحتاجها	0.96 (1.20, ±2)	-1.16	0.36
15. If necessary, I can easily reach care and welfare services by telephone and in person	15. إذا لزم الأمر، يمكنني الوصول بسهولة إلى خدمات الرعاية والرفاه عبر الهاتف أو بشكل شخصيّ	0.74 (1.37, ±2)	-0.86	-0.57
16. I have enough information about care and welfare services in my neighbourhood	16. لديّ معلومات كافية عن خدمات الرعاية والرفاه في الحيّ الذي أسكن فيه	0.07 (1.43, ±2)	-0.15	-1.35
17. Care and welfare workers in my neighbourhood are sufficiently respectful	17. يتصرف موظّفو الرعاية والرفاه الاجتماعيّ في الحيّ الذي أسكن فيه باحترام كافٍ	1.16 (1.01, ±2)	-1.49	2.06
Total		0.68 (0.91)		
Domain 7: Outdoor spaces and buildings	مجال 7: المساحات الخارجية والمباني			
18. My neighbourhood is sufficiently accessible for a wheeled walker or wheelchair	18. الحيّ الذي أسكن فيه متاح بدرجه كافيه لشخص يستخدم مشّاية بعجلات أو كرسيّ متحرك	-1.05 (1.26, ±2)	1.06	-0.29
19. The shops in my neighbourhood are sufficiently accessible with a wheeled walker or wheelchair	19. الوصول إلى الحوانيت في الحيّ الذي أسكن فيه متاح بدرجه كافيه لشخص يستخدم مشّاية بعجلات او كرسيّ متحرك	-1.07 (1.23, ±2)	1.10	-0.15
Total		-1.06 (1.23)		
Domain 8: Transportation	مجال 8: مواصلات			
20. I can easily get on the bus in my neighbourhood.	20. يمكنني بسهولة ركوب الحافلة في الحيّ الذي أسكن فيه	-0.37 (1.53, ±2)	0.36	-1.40
21. The bus stops in my neighbourhood are easy to reach and use.	21. من السهل الوصول إلى مواقف الحافلات في الحيّ الذي أسكن فيه واستخدامها.	-0.39 (1.53, ±2)	0.37	-1.40
Total		-0.38 (1.34)		
Domain 9: Financial situation	مجال 9: الوضع المالي			
22. My income is sufficient to cover my basic needs without any problems	22. مدخولي الشهري يكفي لتغطية احتياجاتي الأساسيّة دون أيّ مشاكل	-0.04 (1.43, ±2)	-0.09	-1.41
23. I live well on my income	23. أنا أعيش بشكل جيّد على دخلي	-0.12 (1.40, ±2)	0.02	-1.35
Total		-0.08 (1.39)		

^1After reversing the item.^

### 4.5 Item distributional statistics

The skewness and kurtosis values for most items ranged between −3 and +3, indicating an acceptable univariate normal distribution. One exception was item #7, which showed a slightly elevated kurtosis value (6.01). Nevertheless, this value remains within a tolerable range and does not substantially deviate from univariate normality. Five participants (2.5% of the total sample, *N* = 202) exceeded the critical Mahalanobis D^2^ value [χ^2^(23) = 51.81, *p* < 0.001] and were, therefore, considered potential multivariate outliers (Hair et al., 2019). In line with recommendations in the literature (Osborne and Overbay, 2004), these cases were retained in subsequent analyses. Given the slight deviation from multivariate normality, the model was estimated using both Maximum Likelihood (ML) and Bootstrapping procedures with 2,000 resamples (Fox, 2015; Nevitt and Hancock, 2001). The results obtained from both approaches were nearly identical, indicating that the ML estimates were robust to minor deviations from multivariate normality. The consistency between estimation methods confirmed the robustness of the data and justified continuing with the subsequent model validation analyses.

### 4.6 Structural validity of the AFCCQ-Arabic

**Figure 2:**
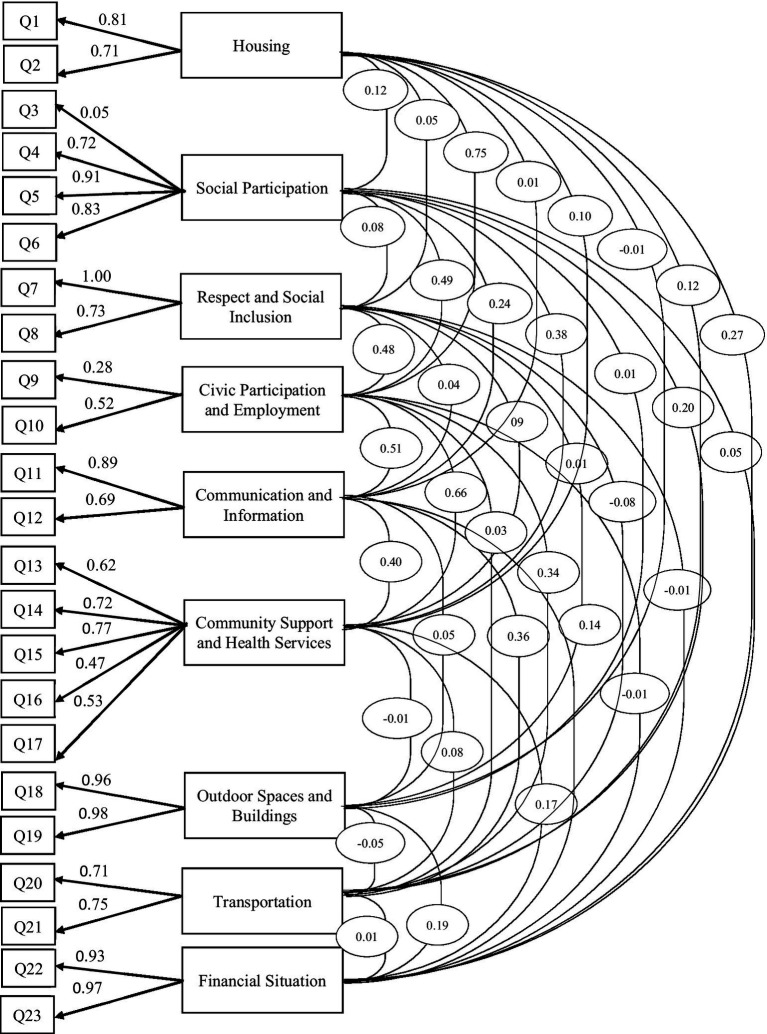
Factor structure based on the confirmatory factor analysis.

The confirmatory factor analysis revealed that most items on the AFCCQ-Arabic demonstrated standardized factor loadings above 0.40, indicating satisfactory associations with their respective latent constructs (see Figure 2). However, two items exhibited lower loadings: item #3 (“opportunities to meet people in the neighborhood”) from the social participation domain (loading = 0.056), and item #9 (“opportunities to interact with younger generations”) from the civic participation and employment domain (loading = 0.280). Despite these lower values, the overall pattern of loadings aligns well with the factor structure proposed by Dikken et al. (2020).

The model fit indices, presented in Table 3, were examined in three stages to assess the stability and adequacy of the factor structure. In the first stage, the full model with all 23 items was tested, yielding acceptable fit indices [χ^2^/df = 1.812, CFI = 0.922, TLI = 0.900, SRMR = 0.072, RMSEA = 0.064 (90% CI: 0.053–0.074)]. In the second stage, item #3, which had a very low factor loading (0.056), was removed, resulting in improved model fit [χ^2^/df = 1.577, CFI = 0.949, TLI = 0.934, SRMR = 0.058, RMSEA = 0.054 (90% CI: 0.041–0.065)]. In the final stage, the entire civic participation and employment domain was excluded, as it consisted of only two items, one of which (item #9) showed a low factor loading (0.280). Although the removal of this domain did not lead to uniform improvement across all fit indices, the final model showed mixed but generally improved fit. Specifically, CFI and TLI increased to 0.955 and 0.941, respectively, while χ^2^/df and SRMR showed slight increases to 1.594 and 0.060. RMSEA remained stable at 0.054 (90% CI: 0.054–0.067). All indices remained within acceptable thresholds, supporting the adequacy of the final model.

**Table 3:** Model fit indices for the AFCCQ-Arabic.

Model	ᵡ^2^/df	P	CFI	TLI	SRMR	RMSEA [90% confidence interval]
All items	1.812	0.000	0.922	0.900	0.072	0.064 [0.053–0.074]
Without item 3	1.577	0.000	0.949	0.934	0.058	0.054 [0.041–0.065]
Without items 3, 9, and 10	1.594	0.000	0.955	0.941	0.060	0.054 [0.054–0.067]

CFI, Confirmatory fit index; TLI, Tucker-Lewis index; SRMR, standardized root-mean-square residual; RMSEA, Root Mean Square Error of Approximation.

### 4.7 Composite reliability

As shown in Table 4, all factors demonstrated CR values above 0.70, indicating acceptable reliability, except for the civic participation and employment factor, which showed a low CR value of 0.282. Notably, the removal of item #3 (from the *Social Participation* domain) led to a marked improvement in reliability, with the CR increasing from 0.769 to 0.868, an increase of nearly 13%. This suggests that item #3 negatively affected the internal consistency of its domain, and its exclusion strengthens the scale’s reliability.

**Table 4:** Composite reliability per factor of the AFCCQ-Arabic.

Factor	Housing	Social participation		Respect and social inclusion	Civic participation and employment	Communication and information	Community support and health services	Outdoor spaces and buildings	Transportation	Financial situation
		All Items	Without item 3							
Composite reliability	0.742	0.769	0.868	0.869	0.282	0.778	0.767	0.976	0.732	0.953

## 5 Discussion

The present study aimed to examine the psychometric properties of the AFCCQ-Arabic and to measure how older Arabs in Israel perceive the age-friendliness of their living environments, within the context of being an ethnic minority facing social, cultural, and political challenges, and undergoing a process of rapid urbanization. Accordingly, the discussion first focuses on evaluating the psychometric validation of the AFCCQ-Arabic, followed by an interpretation of participants’ perceptions across the different age-friendly domains.

### 5.1 Psychometric validation of the AFCCQ-Arabic

The findings generally support the tool’s reliability and validity, indicating an acceptable factor structure and satisfactory internal consistency across most domains, along with strong face and content validity. However, some psychometric concerns emerged, primarily the weak factor loading of Item 3 in the social participation domain and the low internal consistency of the civic participation and employment domain. Item 3, “There are enough opportunities to meet people in my neighborhood,” showed a very weak loading (0.056) and contributed little to the latent construct of social participation, and removing it led to a substantial improvement in the domain’s reliability (from 0.769 to 0.868). This can be explained by the fact that, in Arab society, social participation is often expressed through informal, family- and community-based interactions, such as visiting relatives or attending weddings, rather than through structured programs or civic initiatives. Accordingly, interactions of this kind, “opportunities to meet people in the neighborhood,” typically occur in informal contexts and are not necessarily associated with organized or institutionalized forms of engagement, as reflected in the other items within this domain.

In addition, in the civic participation and employment domain, Item 9, “I have enough opportunities to interact with younger generations,” showed a weak loading (0.28), and the domain exhibited low composite reliability (CR = 0.282). This may indicate that the item does not fully capture the intended construct in this population. A similar pattern was observed in the Albanian-speaking sample in the North Macedonian validation study, where this domain showed low reliability, possibly due to limited engagement in formal employment among older adults (Pavlovski et al., 2024). In the present study, the mismatch may reflect the fact that intergenerational engagement among older Arabs occurs mainly in informal, family-based settings rather than through civic or employment structures. These interactions often involve caregiving and support roles in everyday life (Zakari et al., 2022).

### 5.2 Older Arabs’ perceptions across the AFCC domains

The findings also provide insight into how older Arabs in Israel perceive the age friendliness of their living environments across the AFCC domains. A particularly noteworthy result is the high score reported in the domain of respect and social inclusion (Mean = 1.49), the highest among all domains assessed. This contrasts with lower ratings found among older Jews in Israel (Ayalon et al., 2024). A possible explanation for this finding may stem from cultural and social values in Arab society that emphasize intergenerational respect and the central social status of older adults (AboJabel and Abo-Rass, 2025; Bergman et al., 2013). At the same time, individuals in Arab society tend to act in accordance with accepted social norms and expected roles (Nijm-Akhtilat et al., 2018). Accordingly, it can be assumed that many older adults tend to fulfill traditional age-related roles, such as grandmothers taking an active part in caring for grandchildren, or men serving as sources of social reconciliation or attending the mosque regularly (Findler et al., 2017; Myers-JDC-Brookdale Institute, 2014). By maintaining these socially expected behavior patterns, they may be protected from overt experiences of ageism. In contrast, among older Jews in Israel, recent research points to an expansion beyond traditional age-related roles and the adoption of more innovative aging patterns. These include extended participation in the labor market, civic volunteering, the use of technology to access services, and even active political engagement, such as participation in mass protests (Arad and Reingewertz, 2024; Ayalon and Okun, 2024; Haimi and Sergienko, 2024; Sun and Ayalon, 2024). These trends reflect a more flexible and dynamic approach to age roles, yet they may also expose older adults to different types of ageism. This assumption is consistent with labeling theory (Link and Phelan, 2001), which suggests that individuals who conform to socially expected roles are less likely to be labeled or stigmatized. Therefore, the high sense of respect reported may reflect both the cultural reverence toward older adults and their adherence to socially and culturally defined models of aging.

Another domain that received a particularly high rating in the current study was housing (Mean = 1.00), indicating that most participants perceived their living conditions as age-friendly and accessible. This result is consistent with previous research using the AFCCQ among diverse populations, where housing is consistently ranked as one of the most positively evaluated domains (for instance, Ivan et al., 2024; Pavlovski et al., 2024). In the current sample, 98% of participants were homeowners, and they had lived in their current place of residence for an average of approximately 64 years. These figures likely contribute to the high housing scores, as long-term residence fosters strong familiarity with the home environment, reduces the need for relocation or structural adaptations, and enhances feelings of autonomy and stability. Moreover, remaining in the same home for decades may strengthen emotional attachment to the physical space and neighborhood, which are important aspects of aging in place (Wiles et al., 2012). However, this high level of satisfaction may not extend to future generations. Given the broader housing crisis in Israel, affecting both Arab and Jewish populations, future older adults may face greater challenges in achieving similar housing stability.

The domains of community support and health services (Mean = 0.68) received moderate to relatively high ratings. However, these findings should be interpreted with caution. Among older Arabs in Israel, reported satisfaction with community support may primarily reflect the strong role of the family as the main provider of emotional and instrumental assistance, and not necessarily the accessibility or quality of formal health and welfare services (AboJabel and Abo-Rass, 2025). Nevertheless, some studies suggest that modernization processes may be eroding traditional patterns of family caregiving, contributing to a growing sense of dissatisfaction and concern among older adults regarding the adequacy and consistency of the support they receive (Ayalon, 2018).

The communication and information domain received a moderate score (Mean = 0.08), indicating partial satisfaction alongside existing gaps. One of the main reasons is language barriers, as much of the information provided by public institutions is written in Hebrew, which is often inaccessible to older Arabs. According to the Israeli Central Bureau of Statistics (2019–2021), only 36.5% of Arab citizens speak Hebrew, 30.8% can read it, and just 29.9% can write it. Additionally, an estimated 32.5% of the Arab population have completed only up to 4 years of formal education, which suggests limited literacy skills even in Arabic. Nevertheless, these barriers may be partially mitigated by strong familial support systems, particularly in cases where adult children assist their older parents in accessing, understanding, and navigating written and digital information.

Finally, the lowest-scoring domains in this study, outdoor spaces and buildings (Mean = −1.06), transportation (Mean = −0.38), and financial situation (Mean = −0.08), reflect persistent structural disadvantages in Arab localities in Israel. Research has shown that Arab towns frequently lack accessible and well-maintained public spaces, limiting opportunities for recreation, mobility, and community interaction. Similarly, public transportation tends to be sparse in many semi-urban Arab areas (Khamaisi, 2016; Ministry of Construction and Housing, 2021). Structural and economic barriers partly explain the limited use of public spaces. In addition, cultural factors also play a key role in Middle Eastern societies. Traditionally, these societies emphasize privacy, family life, and modesty, often favoring private over public life. However, recent cultural and social shifts call for a renewed understanding of the public space and its role in Arab social life (Alnaim and Noaime, 2023). Finally, the low score in the financial situation domain reflects the widespread economic vulnerability of older Arabs in Israel. Over 58% live below the poverty line, mainly due to long-term poverty, reliance solely on basic state pensions, and limited job opportunities after retirement (Nathanson et al., 2018).

## 6 Conclusion, implications, and limitations

This study provides initial psychometric support for the AFCCQ-Arabic and offers valuable insights into the experiences of older Arabs in Israel. Despite some limitations, the AFCCQ-Arabic demonstrated overall satisfactory psychometric properties among older Arabs in Israel. Retaining the questionnaire in its current form is recommended to enable meaningful international comparisons. Nonetheless, particular caution should be exercised when interpreting findings related to Item 3 in the social participation domain and the civic participation and employment domain, which showed weaker psychometric performance.

Regarding the lived experiences of older Arabs in Israel, several domains showed relatively high satisfaction, particularly respect and social inclusion and housing, while others, such as outdoor spaces, transportation, and financial situation, revealed areas of concern that reflect structural and economic disparities in Arab localities. These results carry several important implications. At the policy level, they highlight the need for targeted investment in infrastructure and services tailored to the needs of aging minority populations, including public transportation systems, accessible outdoor environments, and financial security programs that take into account the unique socio-economic profile of older Arabs. This is particularly important in light of the housing transitions occurring within the Arab society, where increasing urban density and a gradual shift toward apartment living may further affect older adults’ mobility, access to services, and quality of life. For local municipalities, the AFCCQ-Arabic serves as a culturally adapted and practical tool for assessing AFCC level, enabling identification of service gaps and planning of interventions that resonate with local realities. In addition, it may facilitate more inclusive decision-making processes by encouraging the involvement of older adults as active participants in shaping their living environments.

Despite its contributions, this study has some limitations. First, the recruitment process and sample characteristics limit the generalizability of the findings. The study relied on a convenience sample, and recruiting a probability-based sample of older Arabs in Israel presents considerable methodological and cultural challenges. Regarding the sample characteristics, the composition of the sample, in which women represented the majority (65%), may have influenced the reported perceptions of age friendliness. Previous studies have demonstrated significant gender differences in mobility patterns, social participation, use of public spaces, and service utilization among older adults, reflecting variations in social roles, cultural norms, and environmental accessibility (Gajović et al., 2021; Mitra et al., 2021; Ong et al., 2024; Sütlü and Büyükyörük, 2025; Yu et al., 2021). Therefore, the findings may to some extent reflect women’s perspectives more than men’s, highlighting the need for future research to examine gender differences in perceptions and experiences of age friendliness more specifically. Second, although the data were collected through face-to-face interviews and participants were assured of confidentiality, the possibility of social desirability bias cannot be entirely ruled out. Third, data collection was conducted during a period of security tension and intermittent military escalations, which may have influenced participants’ emotional state, concentration, and response accuracy, as well as their availability and willingness to participate. However, interviews were carried out by experienced and trained staff who were able to identify signs of distress and pause interviews when necessary. Furthermore, data collection was conducted in accordance with Home Front Command safety guidelines, during periods of relative calm, and was suspended during security escalations, which helped minimize potential biases related to the security situation. Fourth, the study focused on a single cultural group, the Arab society in Israel, thus preventing examination of measurement invariance across different cultural or linguistic groups. Future research should therefore include additional populations to assess the cross-cultural validity of the tool. Fifth, the study did not include an examination of criterion validity, as this phase focused on evaluating the internal psychometric properties of the AFCCQ Arabic, including construct validity and reliability. Future studies should extend this work by testing criterion validity to enhance the instrument’s scientific and practical utility. Finally, although the present findings support the validity and reliability of the Arabic version of the questionnaire within the context of Arabs in Israel, further validation is recommended among other Arabic speaking populations, including Arabs living as minorities in non-Arab countries, such as Arab immigrants and communities in Europe or the United States, as well as those residing in Arab countries where cultural, social, and economic contexts differ. In addition, future studies should include larger samples to further strengthen model fit, reliability, and the overall robustness and generalizability of the findings.

## Data Availability

The raw data supporting the conclusions of this article will be made available by the authors, without undue reservation.

## Footnotes

^1^ https://www.oref.org.il/eng
